# Supraphysiological Doses of Vitamin A Promote Changes in Hippocampal Oscillation Bands and Cardiac Activity in Wistar Rats

**DOI:** 10.1021/acsomega.6c01082

**Published:** 2026-05-06

**Authors:** Priscille Fidelis Pacheco Hartcopff, Axell Lins, Daniella Bastos de Araujo, Gabriela Brito Barbosa, Luana Vasconcelos de Souza, Maria Klara Otake Hamoy, Luciana Eiró-Quirino, Silene Maria Araujo de Lima, Nilton Akio Muto, Dielly Catrina Favacho Lopes, Moisés Hamoy

**Affiliations:** † Laboratory of Pharmacology and Toxicology of Natural Products, Biological Sciences Institute, 37871 Federal University of Para, Belém 66075-110, Pará, Brazil; ‡ Neurobiology Laboratory, Biological Sciences Institute, Federal University of Para, Belém 66075-110, Pará, Brazil; § Center of the Valorisation of Amazonian Bioactive Compounds,Federal University of Pará, Belém 66075-110, Pará, Brazil; ∥ Laboratory of Experimental Neuropathology, Joao de Barros Barreto University Hospital, Federal University of Para, Belém 66075-110, Pará, Brazil

## Abstract

Vitamin A is an essential micronutrient involved in growth, immune regulation, and central nervous system function; however, excessive intake may disrupt systemic homeostasis. This study investigated the electrophysiological effects of experimental hypervitaminosis A on hippocampal activity and cardiac function in male Wistar rats. Animals received a supraphysiological dose of vitamin A (50,000 IU/kg, intraperitoneally) once daily for 3, 7, or 14 days. Serum calcium levels were quantified, and electrophysiological recordings were obtained from the CA1 hippocampal region (EEG) and through electrocardiography (ECG). Vitamin A administration produced a time-dependent increase in total hippocampal spectral power. Power elevations were observed across multiple frequency bands, particularly theta (4–8 Hz), α (8–12 Hz), β (12–28 Hz), and γ (28–40 Hz) oscillations after 7 and 14 days of exposure. Serum calcium levels showed modest increases but remained within physiological reference ranges. Cardiac assessment revealed increased heart rate accompanied by reductions in R–R and QT intervals while maintaining sinus rhythm and preserved ECG morphology. These findings demonstrate that short-term exposure to supraphysiological vitamin A levels alters hippocampal oscillatory dynamics and modulates cardiac electrophysiological parameters in rats. Although the functional and mechanistic implications require further investigation, the results indicate that excessive vitamin A intake can influence neurocardiac electrophysiological homeostasis even in the absence of overt hypercalcemia.

## Graphical abstract

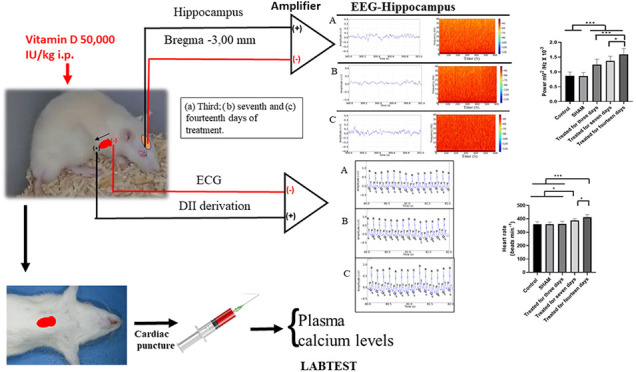

## 1 Introduction

Vitamin A is an essential fat-soluble micronutrient involved in growth, immune regulation, tissue differentiation, and central nervous system (CNS) function.^1–4^ Its biological activity is mediated by retinoids, including retinol and retinoic acid, which regulate gene transcription through nuclear receptors and influence cellular proliferation, differentiation, and metabolic homeostasis.^3,5^ While vitamin A deficiency has been extensively investigated due to its global health relevance,^6–8^ increasing attention has been directed toward the adverse effects associated with excessive intake.^9–13^ Hypervitaminosis A has been linked to skeletal alterations, teratogenicity, intracranial hypertension, and cardiovascular disturbances.^14–16,24–26^

Retinoid signaling components are widely expressed in the adult brain, particularly in the hippocampus, where they participate in synaptic plasticity, neuronal differentiation, and modulation of excitatory transmission.^17,18,39,41–43^ Retinoic acid has been implicated in affective regulation and neuropsychiatric conditions,^39,52^ and alterations in retinoid balance may influence neuronal excitability. Experimental studies indicate that extracellular calcium plays a crucial role in regulating hippocampal firing properties and synaptic responsiveness.^27–30^ Given that vitamin A excess can modify bone metabolism and systemic calcium dynamics,^24–26,36–38^ it is plausible that supraphysiological exposure could indirectly or directly affect neuronal network activity.

Hippocampal oscillations reflect coordinated neuronal synchronization and are sensitive to metabolic and ionic changes.^31,32^ Alterations in spectral power distribution across frequency bands have been described in several neurological and psychiatric disorders.^44–49^ However, oscillatory disturbances are highly context-dependent and do not necessarily indicate specific pathological states. Despite evidence suggesting that retinoids modulate neuronal signaling, there is limited information regarding how experimental hypervitaminosis A influences the in vivo electrophysiological patterns in the hippocampus.

In addition to central effects, vitamin A derivatives have been associated with cardiovascular alterations, including conduction abnormalities and changes in autonomic regulation.^16,61–63^ Cardiac electrophysiology is also sensitive to extracellular calcium concentrations and ionic balance.^33,34^ Nevertheless, most available data derive from clinical observations or studies evaluating isolated parameters, and integrated assessments of central and peripheral electrophysiological markers under conditions of vitamin A excess remain scarce.

From a nutritional and toxicological perspective, excessive vitamin A intake represents a relevant concern due to supplementation practices and over-the-counter availability.^9,13^ Although systemic manifestations of hypervitaminosis A have been described, its impact on coordinated neurocardiac electrophysiological dynamics has not been comprehensively characterized in experimental models.

Therefore, the present study aimed to investigate whether short-term exposure to a supraphysiological dose of vitamin A modifies hippocampal oscillatory activity and cardiac electrophysiological parameters in male Wistar rats. By combining spectral electroencephalographic (EEG) analysis, electrocardiographic (ECG) assessment, and serum calcium quantification, we sought to characterize the functional electrophysiological profile associated with experimental hypervitaminosis A.

## 2 Materials and Methods

### 2.1 Animals

A total of 45 male Wistar rats (*Rattus norvegicus*, aged 8–10 weeks old and weighing 200 ± 20 g) were used for the experiments. The animals were kept in an environment with a temperature of 22 ± 2 °C, an appropriate humidity of around 55 ± 10% relative air humidity, artificial light with a photoperiod of 12/12 h, and with controlled noise. They were housed in cages with filtered water and *ad libitum* access to food throughout the study. The cages were cleaned three times a week. The electrical recordings were made between 8:00 am and 11:00 am.

The study was conducted after approval by the Ethics Committee for Research with Experimental Animals of UFPA (CEPAE-UFPA), protocol no. 2252220321. All of the experimental procedures were conducted in accordance with the principles of the Brazilian National Council for the Control of Animal Experimentation (CONCEA) and performed according to the Animal Research: Reporting In Vivo Experiments (ARRIVE) guidelines.^19^ All necessary precautions were taken to prevent animal suffering and distress.

### 2.2 Drugs

The following chemical substances were used to perform the study: ketamine hydrochloride (Köing Laboratory, Santana de Parnaíba, SP, Brazil), xylazine hydrochloride (Vallée Laboratory, Montes Claros, MG, Brazil), and Arovit (retinol) 300,000 IU/ml (Roche Chemicals and Pharmaceuticals S/A Laboratory, São Paulo - SP, Brazil).

### 2.3 Experimental Design

In this study, doses were administered (50,000 IU/kg) via intraperitoneal injection (i.p.). The dose used in this study was selected based on the experimental model described by Ay and Aslan (2023),^20^ who administered graded doses of vitamin A (10,000–200,000 IU/kg) to Wistar rats. In their study, the 50,000 IU/kg dose produced marked biological effects and represented an intermediate point within the toxic range established by the authors and commonly applied in hypervitaminosis experimental models. The treatment was administered once daily up to 14 days, and the data were recorded on the 3rd, 7th, and 14th day. The selected dose represents a supraphysiological intake scenario commonly used to model hypervitaminosis A.

The animals were divided into groups as follows (*n* = 9 animals/group): (a) CTRL: control group, animals that did not receive any drug; (b) VEH: vehicle group, animals that received physiological saline solution (0.9%, 0.15 mL, i.p.) for 14 days; (c) VA-3d: animals that received vitamin A (50,000 IU/kg, i.p.) for 3 days; (d) VA-7d: animals that received vitamin A (50,000 IU/kg, i.p.) for 7 days; and (e) VA-14d: animals that received vitamin A (50,000 IU/kg, i.p.) for 14 days.

The animals were evaluated for electrophysiological recordings from the CA1 region of the hippocampus (EEG-H) and electrocardiogram.

#### 2.3.1 Surgery for Electrode Implantation

Three days before the start of vitamin A administration, the animals were anesthetized (i.p.) with ketamine hydrochloride (100 mg/kg) and xylazine hydrochloride (5 mg/kg). After the abolition of the interdigital reflex, the animals were positioned in a stereotaxic apparatus, and the skull was exposed to electrode implantation on the brain. Stainless steel electrodes measuring 0.3 mm in diameter and 3.4 mm in length, properly insulated, were positioned in the CA1 area of the hippocampus (stereotaxic coordinates from bregma −3.00 mm and ±1.0 mm lateral and 3.4 mm dorsoventral; Figure 1).^21^ A screw was fixed in the occipital skull, and the electrodes were fixed with self-curing acrylic cement.

**Figure 1:**
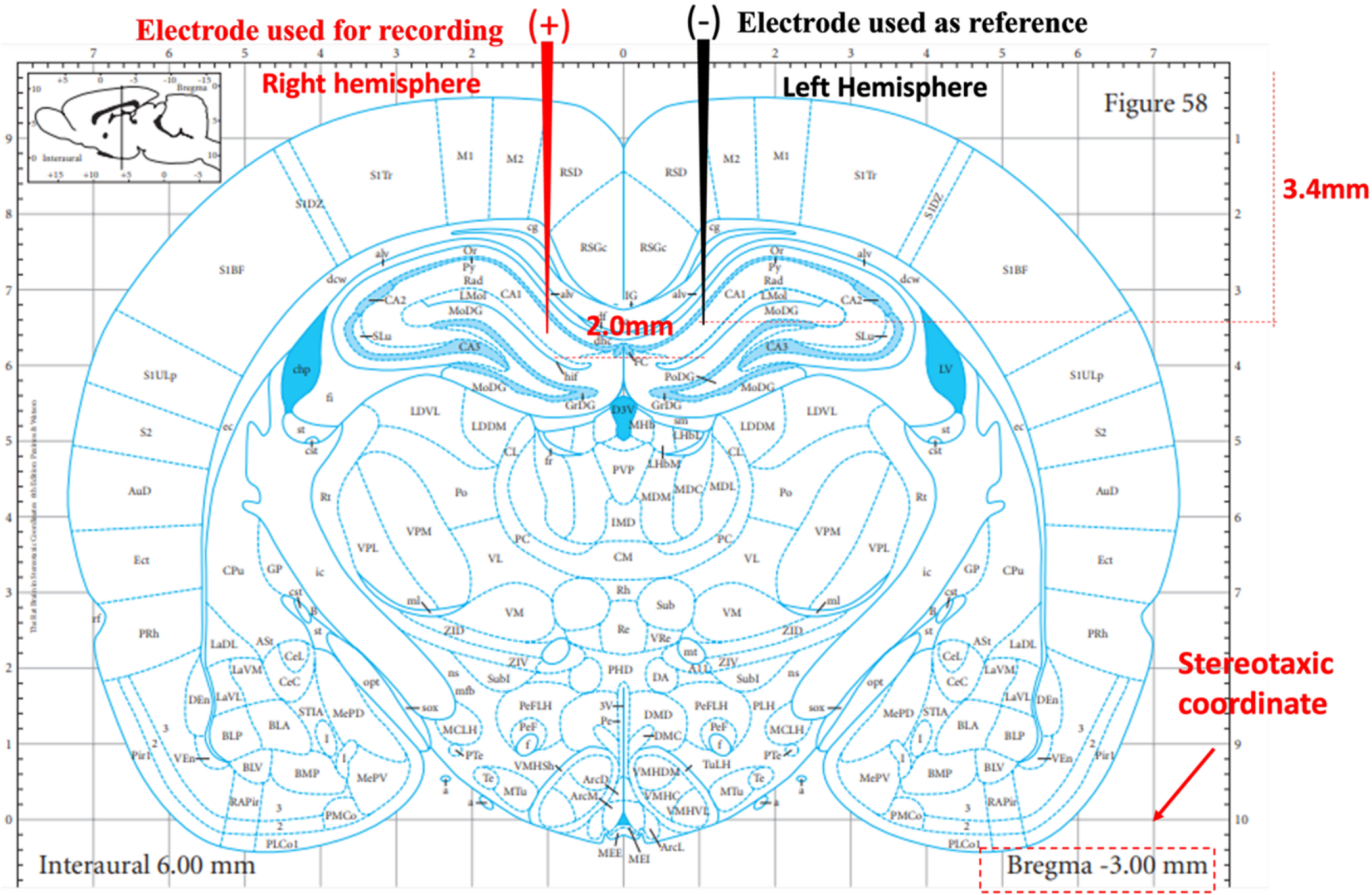
Demonstrates schematic electrode positioning for acquisition of recordings in the hippocampus in the CA1 area, conjugated electrodes 2 mm apart, dorsoventral coordinate of 3.4 mm, electrode on the right side representing the recording electrode, and the electrode on the left side representing the reference electrode.

#### 2.3.2 Recording from the CA1 Region of the Hippocampus (EEG-H)

After electrode implantation, the animals were kept in individual cages until recording. Recording sessions were conducted on the 3rd, 7th, and 14th day after vitamin A administration. Electrodes were connected to a digital data acquisition system consisting of a high-impedance amplifier (Grass Technologies, P511), an oscilloscope (Protek, 6510), and a data acquisition and digitization board (National Instruments, Austin, TX). Data were sampled continuously at 1 kHz with a 1 kHz low-pass filter and a 0.3 Hz high-pass filter. During recordings, animals were confined in Plexiglas boxes with restricted spacing (20 × 45 × 15 cm).^22^ For all treatments, EEG-H recordings followed a standard protocol with 10 min to accommodation, followed by 10 min to recording.

#### 2.3.3 Electrocardiogram (ECG) Recording

On the 3rd, 7th, and 14th day after vitamin A administration, the electrode for cardiac recordings was implanted to capture the lead D-II,^23^ using the right axillary region (third intercostal space) as the reference for the reference electrode. The recording electrode was placed on the left side, 2 cm lateral to the xiphoid cartilage, above the 13th intercostal space. Each recording lasted for 2 min, and data were analyzed: heart rate (beats per minute, bpm), amplitude (mV), R–R interval (milliseconds, ms), P–Q interval (ms), QRS duration (ms), and QT interval (ms).

The ECG recordings were made 30 min after the EEG-H recordings.

#### 2.3.4 Data Analyses

The analyses were performed by using a tool created in the Python programming language (version 2.7). The ″Numpy″ and “Scipy” libraries were used for mathematical processing, and the “matplotlib” library was used for generating graphs and plots. A graphical interface was developed using the PyQt4 library. Spectrograms were calculated by using a Hamming window with 256 points (256/1000 s). The analyses were performed up to a frequency of 40 Hz and divided into bands according as delta wave (1–4 Hz), theta wave (4–8 Hz), α wave (8–12 Hz), β wave (12–28 Hz), and γ wave (28–40 Hz)^22^ to interpret the dynamics during the development of vitamin A intoxication. Data were expressed as mV^2^/Hz × 10^–3^.

### 2.4 Serum Calcium Levels

To assess serum calcium levels, the rats were anesthetized with ketamine and xylazine. Once adequate anesthetic depth was achieved, blood collection was performed by cardiac puncture, and the blood sample was kept refrigerated. Serum calcium levels were measured using a quantitative colorimetric assay (Calcium Liquiform, Labtest, MG, Brazil).

After blood collection, the animals were euthanized with high doses of ketamine hydrochloride (300 mg/kg, i.p.) and xylazine hydrochloride (30 mg/kg, i.p.) to prevent suffering of the experimental animals, following the institutional requirements for euthanasia.

### 2.5 Statistical Analyses

The normality of data was assessed using the Shapiro–Wilk test. Since the residuals were normally distributed, comparisons between groups were performed using one-way ANOVA, followed by the Tukey’s test; if not, comparisons between groups were made by the Kruskal–Wallis test, followed by the Dunn’s test. The mean values were presented with their respective standard deviations (mean ± SD), and the significance level was set as *p < 0.05. All data were analyzed using the GraphPad Prism 10.1 software (GraphPad Software Inc., San Diego, CA).

## 3 Results

### 3.1 Supraphysiological Doses of Vitamin A Slightly Increased Serum Calcium Levels, with Values within the Normal Range

To evaluate the ability of vitamin A to induce hypercalcemia, we analyzed serum calcium levels (H_(4)_ = 32.73, *p* < 0.0001) during the treatment. No difference was observed between control (8.77 ± 0.48 mg/dL) and vehicle (8.92 ± 0.43 mg/dL; *p* > 0.9999) groups. The VA-3d group (9.83 ± 0.68 mg/dL) also was not statistically different from control and vehicle groups (*p* > 0.05, for both comparisons). The VA-7d group (10.45 ± 0.34 mg/dL) presented increased serum calcium levels in comparison with control and vehicle groups (*p* < 0.01, for both comparisons), but it presented similar levels to VA-3d (*p* > 0.9999). The VA-14d group (11.23 ± 1.32 mg/dL) had higher levels than the control and vehicle (*p* < 0.001, for both comparisons); however, it was similar to the VA-3d (*p* = 0.4160) and VA-7d groups (*p* > 0.9999; Figure 2).

**Figure 2:**
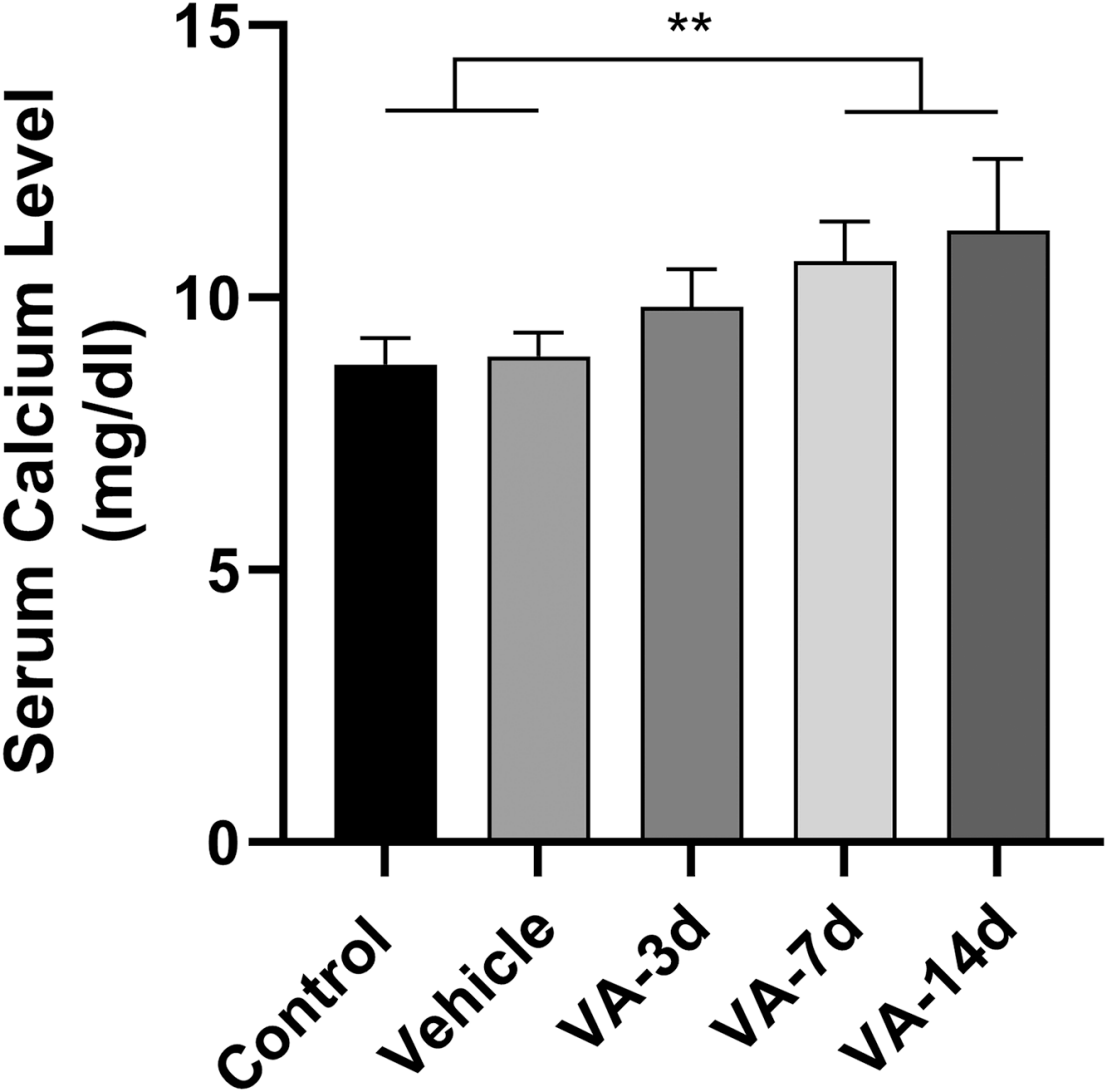
Results of the analysis of serum calcium levels in animals treated or not with vitamin A by a two-way ANOVA test. Data are expressed as mean ± SD (*n* = 9 animals per group, **p < 0.01 and ***p < 0.001).

### 3.2 High Doses of Vitamin A Increased the Amplitude of Brain Waves in the Rat Hippocampus

During the experiment, animals that received supraphysiological doses of vitamin A did not present behavioral changes, and food and water intakes were similar to those of the control and vehicle groups (data not shown).

The EEG-H recordings from the control and vehicle groups presented amplitudes below 0.1 mV (typically low amplitude; Figure 3A,B), as demonstrated in the 1 s amplification (Figure 3A,B, center), and the spectrogram presented the highest power intensity below 10 Hz (Figure 3A,B, right).

**Figure 3:**
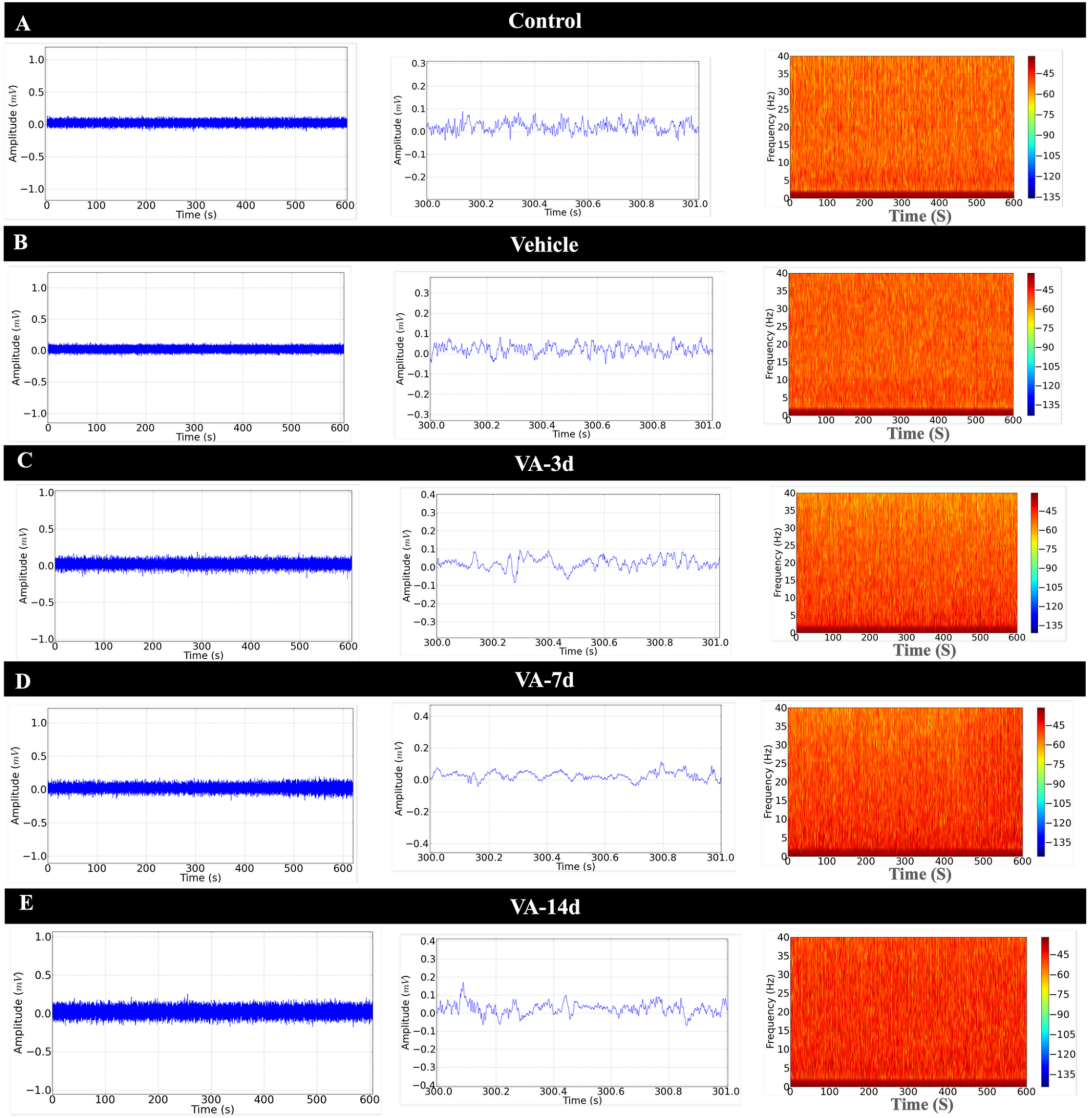
Illustrations of recordings from the rat hippocampus (EEG-H). Tracing of the 10 min hippocampal recording (left), amplification of the recording in 1 s showing amplitude (center) and spectrogram of power distribution at frequencies up to 40 Hz (right), for the following groups: control group (A), SHAM group (B), group treated for 3 days with vitamin A (C), group treated for 7 days with vitamin A (D), and group treated for 14 days (E).

The EEG-H recordings from the VA-3d group presented a greater distribution of power at frequencies above 10 Hz when compared to the control group (Figure 3C). For the VA-7d group, changes in EEG-H traces were observed, which increased the power intensity at frequencies below 30 Hz as shown in the spectrogram (Figure 3D, right). The VA-14d group showed changes in EEG-H traces with greater power at frequencies of up to 40 Hz, with an increase in amplitude at 0.2 Hz (Figure 3E).

The decomposition of the total spectral power distribution revealed changes in power traces in oscillations up to 40 Hz in animals that received supraphysiological doses of vitamin A for 3, 7, and 14 days (F_(4, 40)_ = 35.32, *p* < 0.0001; Figure 4A). No statistical difference was observed between control (0.87 ± 0.125) and vehicle (0.86 ± 0.12) groups (*p* > 0.9999). All groups that received supraphysiological doses of vitamin A presented total spectral power higher than control and vehicle groups (VA-3d: 1.25 ± 0.18; VA-7d: 1.37 ± 0.16; VA-14d: 1.59 ± 0.20; *p* < 0.001, for all comparisons). Among the treated groups, the VA-14d group showed higher elevation than other groups (*p* < 0.05), and no difference was observed between 3 and 7 days of treatment (*p* = 0.4799).

**Figure 4:**
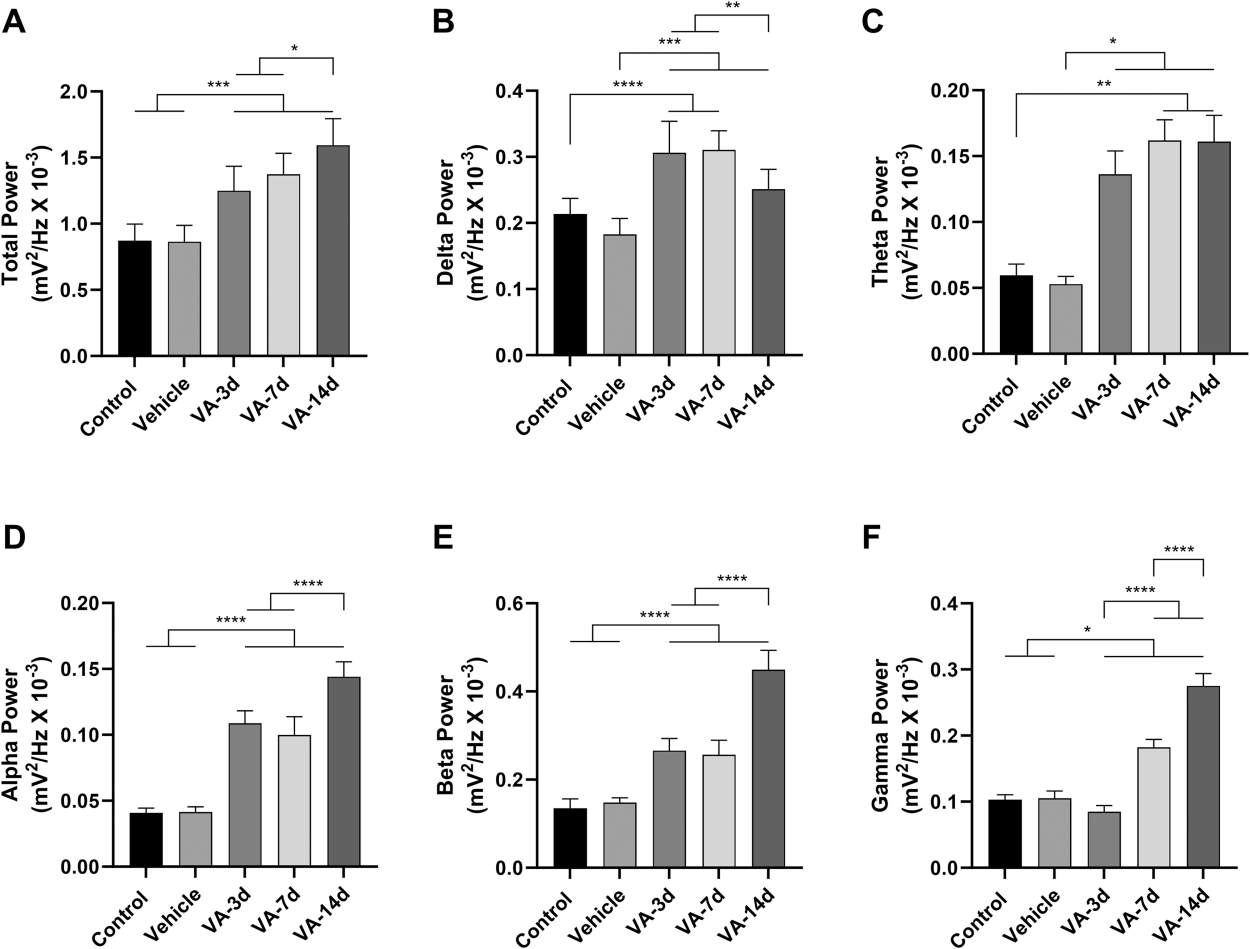
Graph of the linear distribution of power in the oscillations captured in the CA1 area of the rat hippocampus. Showing the average power up to 40 Hz (A). Average power in the delta frequencies (1–4 Hz) (B). Average power in the theta frequency (4–8 Hz) (C). Graph of the linear distribution of power for α oscillations (8–12 Hz) (D). Graph of the linear distribution of power for β oscillations (12–28 Hz) (E). Graph of the linear distribution of power for γ (28–40 Hz) (F) (after ANOVA, followed by Tukey, *p < 0.05, **p < 0.01, ***p < 0.001, *n* = 9).

For frequencies of 1–4 Hz (delta waves; F_(4, 40)_ = 27.55, *p* < 0.0001; Figure 4B) in the hippocampus, no differences were observed between control (0.21 ± 0.024) and vehicle (0.18 ± 0.024) groups (*p* = 0.2703). The administration of vitamin A for 3 and 7 days increased delta wave power in comparison with all other groups (VA-3d: 0.31 ± 0.048; VA-7d: 0.31 ± 0.029; *p* < 0.0001 vs control and vehicle group; and *p* < 0.01 vs VA-14d [0.25 ± 0.030]), but no difference was observed between 3 and 7 days of treatment (*p* = 0.9987).

For theta oscillation frequencies (4–8 Hz; H_(4)_ = 35.62, *p* < 0.0001; Figure 4C) in the hippocampus, we did not observe differences between control (0.060 ± 0.008) and vehicle (0.053 ± 0.006) groups (*p* > 0.9999). One more time, groups that received the higher supraphysiological doses of vitamin A presented theta wave power higher than control and vehicle groups (VA-7d: 0.162 ± 0.016; VA-14d: 0.161 ± 0.020; *p* < 0.01, for all comparisons); and VA-3d (0.136 ± 0.018) showed levels higher than those of the vehicle group (*p* = 0.0386). All groups treated presented similar values to the theta wave.

Both α (8–12 Hz; F_(4, 40)_ = 206.2, *p* < 0.0001; Figure 4D) and β waves frequencies (12–28 Hz; F_(4, 40)_ = 165.0, *p* < 0.0001; Figure 4E) presented similar pattern. No statistical difference was observed between control (α: 0.041 ± 0.004; β: 0.135 ± 0.020) and vehicle (α: 0.041 ± 0.004; β: 0.15 ± 0.01) groups (*p* > 0.05, for both comparisons). For both brain waves, VA-14d (α: 0.144 ± 0.011; β: 0.45 ± 0.04) showed a value higher than other treated groups (VA-3d: α [0.110 ± 0.010] and β [0.27 ± 0.03]; VA-7d: α [0.100 ± 0.014] and β [0.26 ± 0.03]; *p* < 0.0001, for all comparisons). No difference was observed between 3 and 7 days of treatment (*p* > 0.05, for both comparisons to α and β waves).

For the γ oscillations (28–40 Hz; F_(4, 40)_ = 372.7, *p* < 0.0001; Figure 4F), the control group (0.103 ± 0.007) was similar to the vehicle group (0.105 ± 0.010; *p* = 0.9953). Surprisingly, animals treated for 3 days with supraphysiological doses of vitamin A (VA-3d: 0.085 ± 0.010) presented a lower γ wave power than all other groups (*p* < 0.05, for all comparisons). The treatment for 7 days (0.182 ± 0.012) or 14 days (0.275 ± 0.019) showed higher values than other groups (*p* < 0.0001, for all comparisons).

### 3.3 High Doses of Vitamin A Caused Electrocardiographic Changes

The cardiac activity of the control group showed a regular pattern (sinus rhythm), with atrial activity represented by the P wave, ventricular activity represented by the QRS complex, and ventricular repolarization by the T wave (Figure 5A). Animals from the vehicle group presented similar characteristics to the control group, demonstrating cardiac activity in sinus rhythm with all deflagrations of cardiac functioning (Figure 5B). Regarding treated groups with high doses of vitamin A, all of them showed sinus rhythm with maintenance of cardiac electrophysiological characteristics (Figure 5C–E).

**Figure 5:**
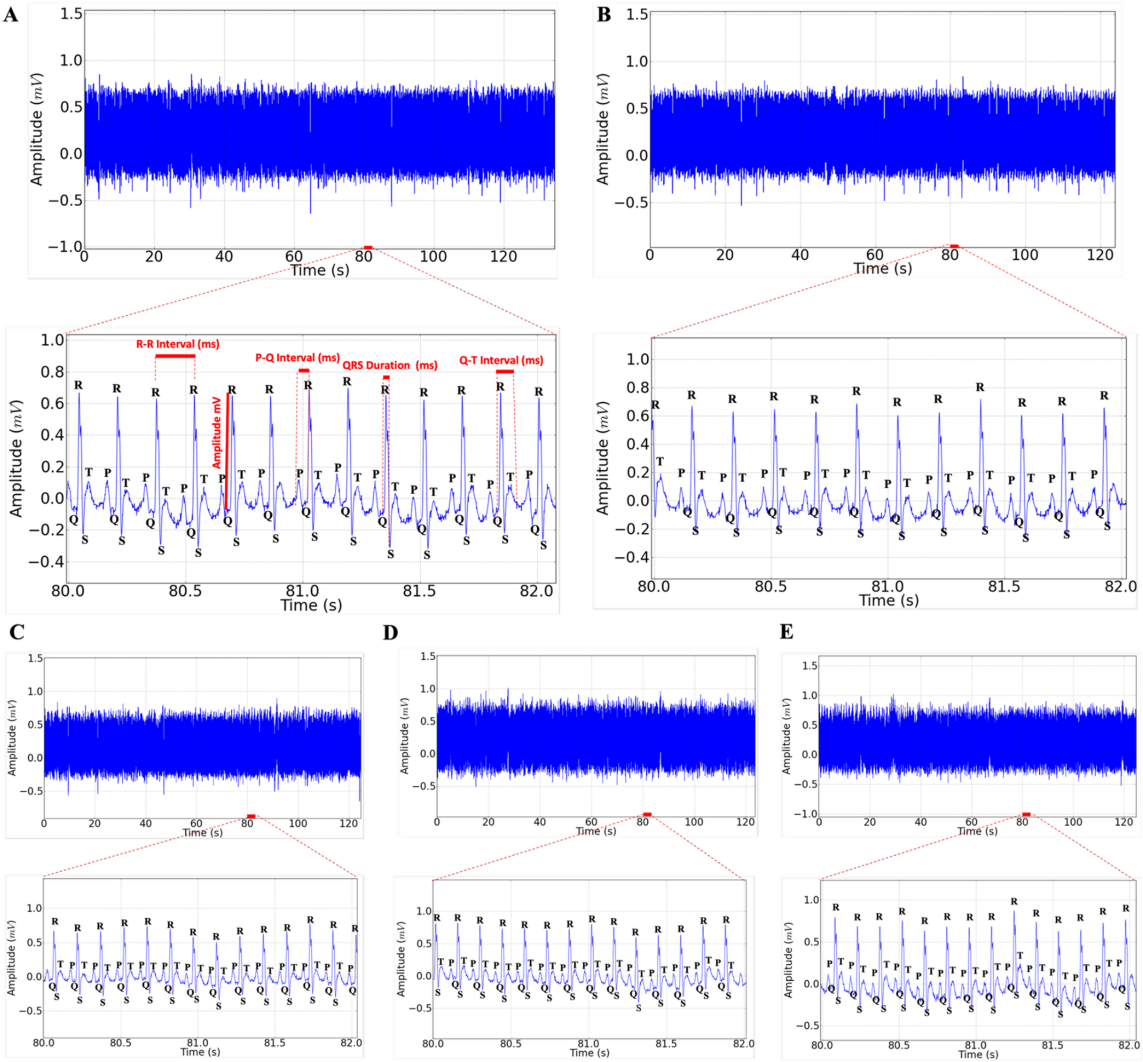
Electrocardiogram in D-II lead lasting 2 min for animals treated for 3 days, amplification of recording in 2 s (recording period 80–82 s) with the presence of P, QRS complex, and T deflagrations for the group treated for 3 days with vitamin A (A). ECG recording represented in the 2 min tracing for the group treated with vitamin A for 7 days, amplification of 2 s recording demonstrating sinus rhythm after 7 days of treatment with vitamin A (B). Electrocardiographic tracing of the animals submitted to treatment with vitamin A for 14 days, amplification of 2 s recording demonstrating the electrocardiographic components after 14 days treatment with vitamin A 50,000 IU/kg i.p. (C).

The cardiac activity was evaluated. For the heart rate, a statistical difference was observed between groups (F_(4, 40)_ = 16.12, *p* < 0.0001; Figure 6A). We did not observe differences between control (360.0 ± 18.28 bpm) and vehicle groups (359.8 ± 15.86 bpm; *p* > 0.9999). The animals that received supraphysiological doses of vitamin A for 14 days (VA-14d group) showed a higher increase in the heart rate average (412.0 ± 19.52 bpm) compared to the control and vehicle groups (*p* < 0.0001, for both comparisons) and in comparison to the VA-3d (362.0 ± 18.52 bpm; *p* < 0.0001) and VA-7d groups (387.6 ± 13.67 bpm; *p* = 0.0355). The VA-7d group also presented increased heart rate in comparison with other groups (*p* < 0.05, vs control, vehicle, and VA-3d groups). No difference was observed between control and vehicle vs VA-3d groups (*p* > 0.05, for both comparisons).

**Figure 6:**
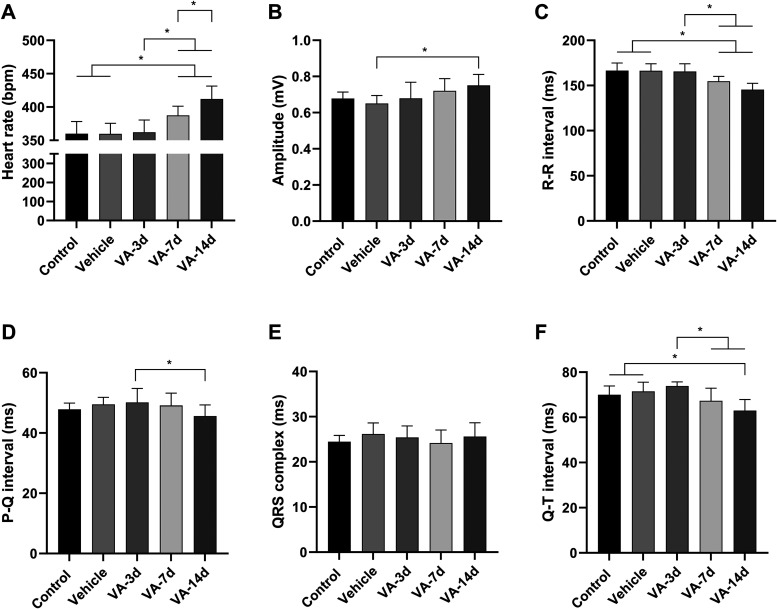
Heart rate averages (bpm) recorded in the control group, SHAM group, after 3 days, after 7 days, and after 14 days of vitamin A treatment (A). Evaluation of the amplitude averages (mV) of electrocardiograms for the groups (B). Evaluation of the R–R interval averages (ms) for the groups (C). P–Q interval averages (ms) recorded in the control, SHAM, after 3 days of treatment, after 7 days of treatment, and after 14 days of vitamin A treatment groups (D). Evaluation of QRS complex duration averages (ms) for the groups (E). Evaluation of the Q–T interval averages (ms) for the groups (F) (after ANOVA, followed by Tukey, *p < 0.05, **p < 0.01, ***p < 0.001, *n* = 9).

The electrocardiogram amplitude (F_(4, 40)_ = 3.662, *p* = 0.0124; Figure 6B) showed a single difference between vehicle (0.651 ± 0.044 mV) and VA-14d groups (0.751 ± 0.060 mV; *p* = 0.0115). For other comparisons, any statistical difference was found (*p* > 0.05, for all comparisons).

About R–R interval (F_(4, 40)_ = 14.49, *p* < 0.0001; Figure 6C), no difference was observed between control (166.6 ± 8.31 ms) and vehicle groups (166.4 ± 7.60 ms; *p* > 0.9999). The VA-14d (145.3 ± 7.02 ms) presented the lower R–R interval between groups (*p* < 0.05, for all comparisons) as well as the VA-7d (154.7 ± 5.48 ms) from other groups (*p* < 0.05, for all comparisons); but no difference was observed between the VA-7d and VA-14d groups (*p* = 0.0796).

For the P–Q interval (F_(4, 39)_ = 2.892, *p* = 0.0345; Figure 6D), a single difference was observed between VA-3d (50.19 ± 4.60 ms) and VA-14d groups (45.62 ± 3.73 ms; *p* = 0.0286). For the other comparisons, any statistical difference was found (*p* > 0.05, for all comparisons). Also, for the QRS interval (F_(4, 40)_ = 0.9462, *p* = 0.4474; Figure 6E), no difference was observed between groups. On the other hand, for the QT interval (F_(4, 40)_ = 2.892, *p* < 0.0001; Figure 6F), no difference was observed between control (70.01 ± 3.89 ms) and vehicle groups (71.47 ± 4.13 ms; *p* = 0.9496), but VA-3d group (73.83 ± 1.88 ms) presented values higher than those of VA-7d (67.30 ± 5.60 ms, *p* = 0.0187) and VA-14d groups (63.02 ± 4.88 ms, *p* < 0.0001). Statistical differences were observed between the VA-14d vs control and vehicle groups (*p* < 0.05, for both comparisons).

## 4 Discussion

This study showed that supraphysiological doses of vitamin A maintained serum calcium levels within the normal range, corresponding to 9.0 to 11.5 mg/dL;^35^ however, it caused changes in the pattern of brain waves, mainly at frequencies above 8 Hz up to 40 Hz (theta, α, β, and γ waves), in addition to increasing heart rate with a reduction in the R–R and QT intervals, but without deformities in the electrocardiographic elements.

In this sense, supraphysiological doses of vitamin A alter bone metabolism, increasing osteoclast activity and, consequently, calcium homeostasis.^24–26,36–38^ However, the results demonstrated that the duration of treatment used showed an increasing variation within the normal range. Extracellular calcium is an important ion that regulates neuronal and cardiac activity.^27^ Changes in Ca^2+^ concentration modify the firing threshold and excitability of hippocampal neurons through calcium-sensitive extracellular receptors and modulation of membrane ion channels.^28–30^ These changes can be observed in the electroencephalogram,^31^ since, in cases of hypercalcemia, the EEG may show rapid activity and bursts of slowing in delta and theta waves.^32^ Calcium also modulates repolarization and the duration of the action potential of the heart muscle;^33,34^ however, during treatment with vitamin A, calcium levels remained within the normal range, showing no interference with the direct effect of vitamin A on hippocampal and cardiac activity; this is the first time that high doses of vitamin A have been reported to affect the functioning of these organs.

Studies have shown that molecular components involved in vitamin A signaling are present in the adult brain, and retinoids have been implicated in affective regulation in some experimental and clinical contexts.^39^ However, under the conditions of our study, no behavioral changes suggestive of affective disorders were observed, such as reduced activity, social withdrawal, or decreased food intake. What makes this type of investigation more difficult is that it requires highly sensitive tools, such as electrophysiology.

Furthermore, excessive intake of vitamin A or its derivatives, such as isotretinoin, has been associated with a variety of central nervous system symptoms in humans.^40^ These reports, however, derive from clinical contexts that differ substantially from those of the experimental conditions of the present study.

In the CNS, retinoids participate in multiple forms of neural plasticity across regions such as the hippocampus, olfactory bulb, and hypothalamus.^18,41–43^ Brain oscillations, however, are influenced by multiple physiological variables, and although retinoic acid can modulate neuronal excitability, there is no direct evidence that excessive vitamin A intake produces specific increases in low-frequency oscillations.^10^ In our study, supraphysiological doses of vitamin A increased the power of low-frequency oscillations (delta, theta, and low-α) in a time-dependent manner. Although oscillations in these frequency bands are involved in cognitive and behavioral processes in humans, their modulation in rodents cannot be directly interpreted as reflecting functional enhancement or impairment. In our study, we demonstrated that supraphysiological doses of vitamin A can increase the power of low-frequency oscillations (delta, theta, and low-α waves) in the CA1 area of the hippocampus of rats in a time-dependent manner.

In addition, in this study, supraphysiological doses of vitamin A increased both low and high-frequency oscillatory power. Although oscillatory disturbances, termed “oscillopathies”, are described in neurological disorders,^44^ such alterations are highly context-dependent and not specific to any disease process.

An increase in delta, theta, and α waves may indicate brain dysfunction,^45^ with α wave activity increasing more predominantly during disorders;^46^ here, the increase in brain wave power occurred more rapidly with oscillations in theta, α, β, and γ, mainly in the group treated for 14 days, but its functional significance cannot be determined in the absence of behavioral or mechanistic assessments, as in these cases, there appears to be a direct effect of vitamin A on the hippocampus.

According to Gaubert et al.,^47^ increase in the power of β and γ waves and a decrease in low-frequency oscillations (delta and theta power) are associated with degenerative processes. After vitamin A administration, an early increase in low-frequency power (delta and theta waves) is observed, which would be contrary to the prevalence of power observed during degenerative processes, as proven by EEG-H. On the other hand, Endres et al.^48^ indicate that a persistent increase in delta and theta power is observed in the EEG of adult patients with attention deficit/hyperactivity disorder. Therefore, supraphysiological doses of vitamin A increased all brain wave oscillations, with a preponderance in theta, α, and β waves in the hippocampus of rats, and the preponderance of the power of these oscillations may alter brain function, worsening according to the time of contact with high doses of vitamin A.

Increasing evidence has shown that psychoactive drugs significantly affect brainwave activity. For instance, tricyclic antidepressants increase activity in the delta, theta, and beta waves during spontaneous EEG, while ketamine, an *N*-methyl-d-aspartate receptor antagonist, increases theta wave power. Additionally, intravenous pentazocine decreases EEG power in the theta, alpha, and beta bands, whereas opioids generally slow down spontaneous EEG by increasing delta wave power. Finally, cannabis essential oil has been shown to significantly increase the average frequency of alpha, theta, and delta waves, alterations that have been associated with irritability, anxiety, and depression;^49^ intravenous pentazocine decreased the EEG power in the theta, α, and β waves,^50^ opioids generally induce a slowing down of spontaneous EEG, increasing the power in the delta wave,^49^ and the cannabis essential oil showed a significant increase in the average frequency of α, theta, and delta waves,^12,51^ associating these increased activities to irritability, anxiety, and depression.^51,52^ These studies showed some similarity to our findings, as vitamin A administration also increased activity in the α, β, delta, and theta bands. These comparisons are purely descriptive, as our type of study does not allow for inferring pathological mechanisms or predicting clinical outcomes. The increase in theta wave can even be described in a beneficial way in the literature,^53–55^ but the increase in other brain waves is associated with pathological conditions, for example, (a) high power of α wave has been linked to neurodegeneration, hippocampal atrophy, onset of Alzheimer’s disease, and epilepsy;^56,57^ (b) the increase in β wave power is also related to epileptogenesis,^58^ and (c) the increase in delta wave power is known to be related to schizophrenia in humans.^59^ Although supraphysiological vitamin A modified the oscillatory activity of hippocampal waves, these findings cannot be interpreted as analogous to the pathological oscillations seen in neurological or psychiatric disorders, given that no disease models were used and no specific biological tests were performed in this study. However, vitamin A increased the proportion of oscillations in the hippocampus.

Moreover, studies have shown that high doses of vitamin A can lead to systemic hypertension, including intracranial hypertension.^60^ In the studies conducted by Ay et al.^61^ and Karadag et al.,^62^ no significant changes were found in the baseline ECG parameters after use of vitamin A derivatives for one month. In addition, Dursun et al.^63^ evaluated the use of isotretinoin, a vitamin A derivative, and found no changes in the QT interval in the ECG. Compared to these studies, our data showed an increase in heart rate with the reduction in R–R and Q–T intervals, without alterations in the P–Q interval and QRS duration; however, sinus rhythm was observed in all recordings.

This study has several limitations that should be considered when the results are analyzed. Although supraphysiological administration of vitamin A produced clear electrophysiological changes, no behavioral or cognitive assessments were performed, which prevents us from establishing whether the changes observed in the EEG translate into functional consequences. In addition, molecular and cellular analyses, such as quantification of retinoid pathway components, synaptic markers, or ion-channel regulation, were not performed, limiting the ability to determine the molecular basis underlying neural and cardiac electrophysiological responses. Another important factor to highlight is that the changes in extracellular calcium were modest and remained within physiological limits, making it difficult to distinguish their contribution from the direct effects related to vitamin A. Therefore, it is not possible to infer the causality between calcium fluctuations and electrophysiological results. The study used only male Wistar rats, and sex-related differences in retinoid metabolism or electrophysiological susceptibility cannot be ruled out. Finally, the short-term exposure model does not allow extrapolation to chronic or clinical scenarios, and the results should not be interpreted as having therapeutic or pathological implications without additional confirmatory studies in disease-specific models. Although the results demonstrate that high doses of vitamin A alter oscillations in the hippocampus of Wistar rats, there are limitations to extrapolating the acute and chronic toxicities of vitamin A in humans.

## 5 Conclusion

Short-term exposure to a supraphysiological dose of vitamin A induced time-dependent modifications in hippocampal oscillatory activity and cardiac electrophysiological parameters in male Wistar rats. Increased spectral power was observed across multiple frequency bands in the CA1 region, while cardiac recordings demonstrated elevated heart rate, accompanied by reductions in R–R and QT intervals, without the disruption of sinus rhythm or major alterations in ECG morphology.

Although serum calcium levels showed modest elevations, they remained within physiological limits, suggesting that the electrophysiological changes observed may not be solely attributable to overt disturbances in the systemic calcium homeostasis.

Taken together, these findings indicate that excessive vitamin A intake can modulate neurocardiac electrophysiological dynamics under experimental conditions. However, the functional and mechanistic implications of these alterations have remained to be elucidated. Further studies incorporating behavioral assessments, molecular analyses of retinoid signaling pathways, and detailed electrophysiological characterization are necessary to clarify the biological significance of these observations and to determine whether similar effects occur under chronic exposure or clinically relevant conditions.

## Data Availability

All data generated or analyzed during this study are available in the public repository at the following link:https://drive.google.com/file/d/11VjxTKyZltmT5N2INltqdcDBuxxaUUCh/view?usp=sharing. The data sets support the findings of this study and include the original electrophysiological and behavioral data.

## References

[1] Blaner W. S. (2019). Vitamin
A signaling and homeostasis in obesity, diabetes, and metabolic disorders. Pharmacol. Ther..

[2] Yee M. M. F., Chin K.-Y., Ima-Nirwana S., Wong S. K. (2021). Vitamin A and bone
health: A review on current evidence. Molecules.

[3] Conaway H. H., Henning P., Lerner U. H. (2013). Vitamin
A metabolism, action, and
role in skeletal homeostasis. Endocr. Rev..

[4] Zinder R., Cooley R., Vlad L. G., Molnar J. A. (2019). Vitamin A and wound
healing. Nutr. Clin. Pract..

[5] Szymański Ł., Skopek R., Palusińska M. (2020). Retinoic acid and its
derivatives in skin. Cells.

[6] Underwood B. A. (1998). Vitamin
A deficiency. Bull. World Health Organ..

[7] Wirth J., Petry N., Tanumihardjo S. (2017). Vitamin A supplementation
programs and country-level evidence of vitamin A deficiency. Nutrients.

[8] Song A., Mousa H. M., Soifer M., Perez V. L. (2022). Recognizing vitamin
A deficiency: Special considerations in low-prevalence areas. Curr. Opin. Pediatr..

[9] Penniston K. L., Tanumihardjo S. A. (2006). The acute
and chronic toxic effects of vitamin A. Am.
J. Clin. Nutr..

[10] Nisar M., Mohammad R. M., Arshad A. (2019). Influence of dietary
intake on sleeping patterns of medical students. Cureus.

[11] Binkley N., Krueger D. (2009). Hypervitaminosis A
and bone. Nutr. Rev..

[12] de
Oliveira M. R., da Rocha R. F., Pasquali M. A. B., Moreira J. C. F. (2012). Effects
of vitamin A supplementation for 3 months on the adult rat nigrostriatal
axis. Brain Res. Bull..

[13] Sammartano A., Bertoncini L., Barbuti A., Ippolito L. (2023). A suspected hypervitaminosis
A. Acta Biomed..

[14] Nau H. (2001). Teratogenicity
of isotretinoin revisited. J. Am. Acad. Dermatol..

[15] Chagas M. H. d. C., Flores H., Campos F. d. A. C. (2003). Teratogenia da vitamina
A. Rev. Bras. Saúde Mater. Infant..

[16] Shih C.-H., Wu H.-B. (2015). All-trans retinoic
acid–induced complete atrioventricular
block. J. Chin. Med. Assoc..

[17] McCaffery P., Zhang J., Crandall J. E. (2006). Retinoic acid signaling
in the adult
hippocampus. J. Neurobiol..

[18] Shearer K. D., Stoney P. N., Morgan P. J., McCaffery P. J. (2012). A vitamin
for the brain. Trends Neurosci..

[19] Kilkenny C. (2010). Improving bioscience
research reporting: The ARRIVE guidelines. PLoS
Biol..

[20] Ay H., Aslan D. (2023). Effect of retinyl palmitate
on placental volume in rats. Health Sci. Q..

[21] Franklin, K. B. J. ; Paxinos, G. The Mouse Brain in Stereotaxic Coordinates, Compact ed.; Academic Press: London, 2019.

[22] Hamoy M. (2018). Cunaniol-elicited seizures. Toxicol.
Appl.
Pharmacol..

[23] Muto N. A., Hamoy M. (2021). Electrocardiographic
changes caused by açai
stone extract. Toxicol. Rep..

[24] Lips P. (2003). Hypervitaminosis
A and fractures. N. Engl. J. Med..

[25] Lind T. (2017). Vitamin A negatively regulates osteoblast mineralization. PLoS One.

[26] Bøgh-Andersen B. (2022). Hypercalcemia and vitamin A. Cleveland Clin.
J. Med..

[27] Lu B., Su Y., Das S., Liu J., Xia J., Ren D. (2010). The neuronal
NALCN channel contributes to resting sodium permeability and is modulated
by extracellular calcium. Neuron.

[28] Yue C., Yaari Y. (2008). Axo-somatic and apical dendritic action potential initiation
in rat
CA1 pyramidal neurons. J. Neurophysiol..

[29] Aivar P., Valero M., Bellistri E., Martín del Río R., de la Prida L. M. (2014). Extracellular
calcium controls the expression of two
plasticity-related intrinsic currents in hippocampal CA1 pyramidal
neurons. J. Neurosci..

[30] Jones K. A., Smith A. J. (2016). The calcium-sensing
receptor: A potential target for
therapeutics in the central nervous system. Front. Physiol..

[31] Sutter R., Kaplan P. W. (2018). Electroencephalographic
criteria for metabolic encephalopathy. J. Clin.
Neurophysiol..

[32] Nardone R., Brigo F., Trinka E. (2016). Acute symptomatic
seizures caused
by electrolyte disturbances. J. Clin. Neurol..

[33] Passini E., Severi S. (2012). Effects of extracellular
calcium on human ventricular
action potential duration: A computational study. Comput. Cardiol..

[34] Bartolucci C., Severi S., Rodriguez B. (2020). Effects of extracellular calcium
changes on ventricular action potential and its variability in human
and guinea pig cardiomyocytes: A simulation study. Front. Physiol..

[35] Melo, M. G. D. ; Doria, G. A. A. ; Serafini, M. R. ; Araujo, A. A. S. Reference values of hematological and Biochemical Rats (Rattus norvegicus Wistar strain) from the vivarium Central at Federal University of Sergipe Scientia Plena 2012; Vol. 8 4(a), Retrieved from https://scientiaplena.emnuvens.com.br/sp/article/view/494.

[36] Lind T., Lind P. M., Jacobson A. (2011). High dietary intake
of retinol leads to bone marrow hypoxia and diaphyseal endosteal mineralization
in rats. Bone.

[37] Vu A. A., Kushram P., Bose S. (2022). Effects of vitamin
A (retinol) release
from calcium phosphate matrices and porous 3D printed scaffolds on
bone cell proliferation and maturation. ACS
Appl. Bio Mater..

[38] Santos H. B. (2004). Biochemical and hematological
studies in rats on the bioavailability
of minerals in a diet enriched with a multimix. Cienc. Tecnol. Aliment..

[39] Bremner J. D., McCaffery P. (2008). The neurobiology
of retinoic acid in affective disorders. Prog.
Neuropsychopharmacol. Biol. Psychiatry.

[40] Snodgrass S. R. (1992). Vitamin
neurotoxicity. Mol. Neurobiol..

[41] Dowling J. E. (2020). Vitamin
A: Its many roles—from vision and synaptic plasticity to infant
mortality. J. Comp. Physiol. A.

[42] Ashton A., Clark J., Fedo J. (2023). Retinoic acid signalling
in the pineal gland is conserved across mammalian species and its
transcriptional activity is inhibited by melatonin. Cells.

[43] Stoney P. N., McCaffery P. (2016). A vitamin
on the mind: New discoveries on control of
the brain by vitamin A. Nestle Nutr. Inst. Workshop
Ser..

[44] Sahu M., Ambasta R. K., Das S. R. (2024). Harnessing brainwave
entrainment: A non-invasive strategy to alleviate neurological disorder
symptoms. Ageing Res. Rev..

[45] Grin-Yatsenko V. A., Ponomarev V. A., Pronina M. V. (2017). Local and widely distributed
EEG activity in schizophrenia with prevalence of negative symptoms. Clin. EEG Neurosci..

[46] Newson J. J., Thiagarajan T. C. (2019). EEG frequency
bands in psychiatric disorders: A review
of resting-state studies. Front. Hum. Neurosci..

[47] Gaubert S., Raimondo F., Houot M. (2019). EEG evidence of compensatory
mechanisms in preclinical Alzheimer’s disease. Brain.

[48] Endres D. (2017). Increased rates of intermittent rhythmic delta and theta activity
in adult patients with attention-deficit hyperactivity disorder. Epilepsy Behav..

[49] Malver L. P., Brokjær A., Staahl C. (2014). Electroencephalography
and analgesics. Br. J. Clin. Pharmacol..

[50] Bromm B., Ganzel R., Herrmann W. M., Meier W., Scharein E. (1986). Pentazocine
and flupirtine effects on spontaneous and evoked EEG activity. Neuropsychobiology.

[51] Gulluni N., Re T., Loiacono I. (2018). Cannabis essential oil: A preliminary
study for the evaluation of the brain effects. Evidence-Based Complementary Altern. Med..

[52] O’Reilly K., Bailey S. J., Lane M. A. (2008). Retinoid-mediated
regulation of mood:
Possible cellular mechanisms. Exp. Biol. Med..

[53] McNaughton N., Ruan M., Woodnorth M. (2006). Restoring
theta-like rhythmicity
in rats restores initial learning in the Morris water maze. Hippocampus.

[54] Mitchell D. J., McNaughton N., Flanagan D., Kirk I. J. (2008). Frontal-midline
theta from the perspective of hippocampal “theta”. Prog. Neurobiol..

[55] Tan E. (2024). Theta activity and cognitive
functioning: Integrating evidence from
resting-state and task-related developmental EEG research. Dev. Cogn. Neurosci..

[56] El-Hassar L., Esclapez M., Bernard C. (2007). Hyperexcitability
of the CA1 hippocampal
region during epileptogenesis. Epilepsia.

[57] Moretti D. V. (2011). Volumetric differences
in mapped hippocampal regions correlate with
increase of high alpha rhythm in Alzheimer’s disease. Int. J. Alzheimer’s Dis..

[58] Song D. Y., Stoyell S. M., Ross E. E. (2019). Beta oscillations in
the sensorimotor cortex correlate with disease and remission in benign
epilepsy with centrotemporal spikes. Brain Behav..

[59] Zhang Y., Yoshida T., Katz D. B., Lisman J. E. (2012). NMDAR antagonist
action in thalamus imposes delta oscillations on the hippocampus. J. Neurophysiol..

[60] Yehya A., Baer J. T., Smiley W., Dollar A., Sperling L. (2009). Hypervitaminosis
A altering the lipid profile in a hypercholesterolemic patient. J. Clin. Lipidol..

[61] Ay H., Aksoy M., Güngören F. (2019). Assessment
of autonomic
nervous system functions and cardiac rhythms in patients using isotretinoin. Adv. Dermatol. Allergol..

[62] Karadag A. S. (2012). Effects of isotretinoin therapy on electrocardiography, heart rate
and blood pressure. J. Dermatol. Treat..

[63] Dursun R. (2011). Isotretinoin does not
prolong QT intervals and QT dispersion in patients
with severe acne. J. Drugs Dermatol..

